# Effect of Bentonite Pre-Treatment on Growth Performance, Mineral Enrichment, and Antioxidant Properties of Soybean Sprouts

**DOI:** 10.3390/foods15020285

**Published:** 2026-01-13

**Authors:** Mi-Ok Kim, Il-Doo Kim, Mee-Jung Kim, Arjun Adhikari, Jeong-Ho Kim

**Affiliations:** 1 Department of Food and Nutrition, Daegu Health College, Daegu 41453, Republic of Korea; mokim@dhc.ac.kr; 2 Jung Won Food Ltd., Changnyeong-gun 50317, Republic of Korea; ildookim@hanmail.net; 3 Department of Hotel Culinary Art and Baking Science, Gumi University, Gumi 39213, Republic of Korea; cocoa@gumi.ac.kr; 4 Department of Plant Biosciences, Kyungpook National University, Daegu 41566, Republic of Korea; farmerarjun7@gmail.com; 5 Department of Green Technology Convergence, Konkuk University, Chungju 27478, Republic of Korea

**Keywords:** bentonite, sprout yield, nutritional enhancement, isoflavone, antioxidant activity, amino acid

## Abstract

Bentonite is a multifunctional material widely used in industry, agriculture, food processing, and medicine due to its strong binding and absorption properties. This study investigates the effects of bentonite pre-treatment at different concentrations (0.5–5%) on soybean sprout growth and nutritional quality. Moderate levels, particularly 1–3% (BP-1 and BP-3), significantly increased sprout yield (up to 16.1%) and vitamin C content (up to 18.91 mg/100 g FW), while maintaining desirable moisture and visual quality. Color evaluation showed higher yellowness (b*), suggesting improved consumer appeal. Mineral profiling indicated substantial enhancement of essential minerals across treatments, with the highest total mineral content recorded in the BP-5 group. Phosphorus, potassium, copper, and iron were notably enriched; however, elevated copper and reduced zinc at higher concentrations indicate potential nutritional risk. Isoflavone analysis revealed increased total isoflavones, especially glucoside forms such as daidzin and genistin, while aglycones like genistein decreased, reflecting bentonite’s selective influence on isoflavone metabolism. Antioxidant properties—including DPPH scavenging capacity, total polyphenols, flavonoids, and SOD-like activity—were significantly enhanced. Amino acid profiling also showed increases in essential amino acids, including methionine and valine, along with higher γ-aminobutyric acid (GABA). Overall, bentonite demonstrates strong potential as a natural additive for improving soybean sprout productivity and functional quality, with the most favorable outcomes observed at 1–3% concentrations.

## 1. Introduction

Microgreens and sprouts have emerged as highly valued functional foods due to their nutritional profile, rapid production cycle, and suitability for urban and indoor cultivation systems [1,2]. As urban farming expands through hydroponics, vertical farms, and household cultivation, there is increasing interest in natural, low-cost treatments that can further improve the nutritional properties and yield of microgreens [3]. In South Korea, soybean microgreens are increasingly incorporated into traditional and modern dishes such as kimchi variations, salads, bibimbap, and health-focused side dishes, reflecting a growing consumer preference for fresh, nutrient-rich, and visually appealing ingredients [4]. Soybean sprouts, in particular, hold deep cultural and culinary significance in Asian cuisine such as Korea, Japan, and China, being widely used in dishes like kongnamul-guk, bibimbap, and fermented preparations [4,5]. Enhancing their high vitamin, mineral, antioxidant, and isoflavone content could make them an important value-added food for humans, livestock, and economy, such as biofuel and edible oil production [6,7,8,9].

Among these, bentonite—a montmorillonite-rich clay with strong adsorption, ion-exchange, and water-retention capacities—has gained attention in agricultural and food science research. Bentonite is already used for improving soil fertility, purifying food products, and supporting sustainable farming practices, with stress resilience making it a promising candidate for microgreen enhancement [5].

External seed pre-treatment is a widely adopted strategy to enhance germination, sprout value, and stress tolerance [10,11]. Antioxidant activity, mineral nutrient composition, amino acid profile, color attributes, vitamin C content, and yield were selected as key parameters to comprehensively assess sprout quality and stress resilience [12,13]. Antioxidant capacity and antioxidant constituents, including polyphenols, flavonoids, and superoxide dismutase (SOD), play a critical role in scavenging reactive oxygen species generated during abiotic stress, from germination through subsequent stages of plant development. Mineral nutrients serve as essential structural and functional components of proteins, amino acids, and vitamins, contribute to ionic homeostasis in crops, and are indispensable for human nutrition. Additionally, color appearance is an important quality trait influencing consumer acceptance and market preference of sprouts and microgreens [14,15].

Among innovative strategies gaining attention is the use of bentonite powder, a naturally occurring clay mineral characterized by its high cation exchange capacity (CEC) and exceptional water-holding properties [16]. Traditionally employed as a soil conditioner, bentonite’s potential extends beyond soil amendment to direct seed enhancement for legumes such as soybean (*Glycine max*), a globally important crop valued for protein-rich oilseed production [17]. A primary mechanism through which bentonite benefits seed quality is its ability to retain moisture in the immediate seed microenvironment. Bentonite’s hygroscopic nature creates a hydrated “microcosm” around the seed, facilitating water uptake even under water-limited conditions and hence used for seed coating [17,18]. By analyzing changes in yield, mineral composition, isoflavones, antioxidants, and amino acids under different bentonite concentrations, current study aims to clarify the potential of bentonite as a natural enhancer for producing premium-quality microgreens used for modern Korean dietary applications and sustainable food systems.

## 2. Materials and Methods

### 2.1. Experiment Materials and Reagents

Soybean (*Glycine max* L.) seeds of cultivar ‘Sowonkong’ were purchased from a local store in Deagu, Republic of Korea. The mean seed weight was 12 g of 100 seeds. The source of bentonite was applied through the supplement GREAT PLAINS Bentonite Yerba Prima Bentonite Clay Plus Herbal Detox (Yerba Prima Inc., 740 Jefferson Ave., Ashland, OR, USA: Details on Appendix A). The following chemicals and reagents were obtained for the present study: 1,1-diphenyl-2-picrylhydrazyl (DPPH), Folin–Ciocalteau reagent, isoflavone standards (≥95% purity, Sigma-Aldrich Corporation, St. Louis, MO, USA), dimethyl sulfoxide (DMSO), pyrogallol (Sigma-Aldrich Corporation, St. Louis, MO, USA), and amino acid standards (Wako Pure Chemical Industries, Ltd., Osaka, Japan). All the other chemicals were of analytical grade.

### 2.2. Cultivation of Soybean Sprouts and Sprout Yield

Sprouts were grown following the method described by Kim et al. [19], with some modifications. One kilogram of intact seeds (for each treatment and replication) was carefully washed with tap water separately for surface cleaning. The seeds were steeped in tap water containing different amounts of bentonite powder or tap water alone for 8 h. Treatment includes control (seeds soaked in tap water for 8 h), BP-0.5 (seeds soaked in water containing 0.5% (*w*/*v*) bentonite powder for 8 h), BP-1 (seeds soaked in water containing 1.0% (*w*/*v*) bentonite powder for 8 h), BP-3 (seeds soaked in water containing 3% (*w*/*v*) bentonite powder for 8 h), and BP-5 (seeds soaked in water containing 5.0% (*w*/*v*) bentonite powder for 8 h). After soaking, the seeds were kept in 15 L plastic buckets with a perforated base for the sprout cultivation. The seeds and sprouts were watered with two hoses of 1 cm diameter for 2 min every 3 h. Soybean sprouts were grown at room temperature, 24 ± 2 °C, for 6 d. Sprout sample powders were prepared for physicochemical studies. The fresh sprouts were kept at −70 °C and subjected to freeze drying. The freeze-dried sprouts were powdered using a commercial grinder (HIL-G-501, Hanil Co., Ltd., Seoul, Republic of Korea) and strained using a 100-mesh sieve. The samples were kept in airtight sample bottles and stored at −20 °C until analyses. The fresh yield of soybean sprouts was measured by deducting the weight of the empty bucket from the weight of each bucket containing sprouts. Yield was measured after 6 days.

### 2.3. Determination of Moisture and Vitamin C Content

The moisture content of soybean sprouts was determined using the oven-dry method, following the method of AOAC [20], as described by Kim et al. [21], with some modifications. Fresh sprouts (5.0 g) were oven dried until constant weight. After drying, the moisture content was calculated using the following formula: where Wb = weight (g) of sprout before drying and Wa = weight (g) of sprout after drying.

The vitamin C content of sprouts was determined following a standard method [20]. Five grams of sample powder was mixed with 7.5 mL of 3% metaphosphoric acid solution and homogenized (AM-8, Nihonseike Kaisha, Tokyo, Japan), followed by the addition of 12.5 mL of the acid solution and filtration. Six milliliters of the filtrate were titrated with 0.025% of 2,6-dichloroindophenol. In this reaction, the vitamin C contained in the extract is oxidized and the indophenol dye is reduced to a colorless compound.

### 2.4. Color Measurement

L* (lightness), a* (redness, + or greenness, −), and b* (yellowness, + or blueness, −) values of sample powders were measured using a Chroma meter (CR-300, Minolta Corp., Osaka, Japan). A Minolta calibration plate (YCIE = 94.5, XCIE = 0.3160, YCIE = 0.330) and a HunterLab standard plate (L* = 97.51, a* = −0.18, b* = +1.67) were used to standardize the instrument using a D65 illuminant (Biobase, Jinan, China). Color values were measured on three zones of powder sample and mean values were calculated.

### 2.5. Analysis of Free Amino Acid Content

Free amino acids were analyzed following the procedure of Je et al. [22] with some modifications. One gram of sprout sample was hydrolyzed with 6 N HCl (10 mL) in a sealed-vacuum ampoule at 110 °C for 24 h. The HCl was removed from the hydrolyzed sample on a rotary evaporator; the content was mixed with 0.2 M sodium citrate buffer (pH 2.2) to make a volume of 5.0 mL. The mixture passed through a C-18 Sep Pak (Waters Co., Milford, MA, USA) cartridge and was filtered through a 0.22 μm membrane filter (Millipore, Billerica, MA, USA). Amino acids were determined using an automatic amino acid analyzer (Biochrom-20, Pharmacia Biotech Co., Uppsala, Sweden).

### 2.6. Quantification of Mineral Content

Mineral content was determined following the method of Skujins [23] with some modifications. Sample powder (0.5 g) and HNO3 (15.0 mL) were mixed into a cup at 110 °C. The mixture was diluted with equal volume of distilled water. Mineral concentrations were determined using inductively coupled plasma atomic emission spectrometer (ICP AES: Varian Vista, Varian Australia, Victoria, Australia).

### 2.7. Measurement of Isoflavone Content

Isoflavones were measured using High Performance Liquid Chromatography (HPLC) following the procedure described by Jiao et al. [24]. Sample powder (0.2 g) was extracted with 6.0 mL of 80% methanol by ultrasonic-assisted method at 40 °C for 30 min and centrifuged. The supernatant was filtered through a 0.45 µm membrane filter (Millipore) before HPLC analysis. The isoflavones were analyzed under the following conditions of HPLC: flow rate 1 mL/min; the mobile phase—solvent A aqueous acetic acid (0.1%), and solvent B acetic acid in acetonitrile (0.1%). HPLC running condition consisted of a gradient of 13–35% B during a 52 min period; oven temperature was 35 °C. The injection volume was 20 µL. The eluted isoflavones were detected at 260 nm. Each peak was identified by the retention time and the characteristic UV spectrum in comparison with the corresponding standards.

### 2.8. Analysis of Antioxidant Activity, Total Flavonoid, and Total Phenolic Content

DPPH radical-scavenging activity, SOD-like activity, and total phenolic content of the samples were measured using standard protocols with minor modifications. For DPPH, 1 g of sample was extracted in 10 mL methanol (150 rpm, 25 °C, 8 h), centrifuged, and filtered (0.2 µm). Sample extract (0.1 mL) was mixed with an equal volume of 0.1% methanolic DPPH, incubated in the dark for 30 min, and absorbance recorded at 517 nm. SOD-like activity was determined by homogenizing 0.5 g sprout powder in 5 mL phosphate buffer (pH 7.8), centrifuging, and incubating 100 µL supernatant with 1300 µL Tris-HCl buffer and 100 µL 7.2 mM pyrogallol for 10 min in the dark; the reaction was terminated with 50 µL 1 N HCl and absorbance measured at 420 nm. Total phenolic content was estimated using the Folin–Ciocalteau method by reacting 50 µL extract with 250 µL Folin–Ciocalteau reagent and 750 µL 20% Na_2_CO_3_, adjusting the volume to 5 mL, incubating for 2 h at 25 °C in the dark, and reading absorbance at 760 nm. For the TFC determination procedure, the sample extracts (100 μL), absolute methanol (500 μL), 10% AlCl3 (50 μL), 1 M HCl (50 μL), and distilled water (300 μL) were combined in microtubes and incubated. The quercetin equivalent (QE) was used to express the flavonoid content using microplate spectrophotometer (Multiskan GO; Thermo Fischer Scientific, Waltham, MA, USA) at 510 nm. DPPH scavenging activity and SOD-like activity were calculated relative to appropriate controls, and phenolic content was expressed as μg gallic acid equivalents (GAE)/g dry sample [25].

### 2.9. Statistical Analysis

Data were subjected to analysis of variance (ANOVA) using SAS 9.3 (SAS Institute, Cary, NC, USA), and significant differences between means at 5% probability were analyzed using the Tukey test. Average values of triplicate measurements were considered for statistical analysis unless otherwise mentioned.

## 3. Results

### 3.1. Effect on Yield, Moisture Level, and Vitamin C Content of Soybean Sprout

Treatment with bentonite powder markedly influenced the total weight, moisture content, and vitamin C levels of soybean sprouts compared to the control. Total weight increased progressively with bentonite concentration, with BP-0.5, BP-1, BP-3, and BP-5 showing 2.2%, 10.3%, 16.1%, and 4.2% higher biomass than the control, respectively; the highest value was recorded in BP-3 (6588 g), which was significantly superior to all other treatments. Moisture content remained statistically similar among all bentonite treatments (86.98–87.41%), although all were substantially higher than the control (86.99%). Vitamin C content also improved with increasing bentonite concentration, with BP-1 and BP-3 exhibiting the greatest enhancement at 17.4% and 18.1% above the control, respectively. BP-5 showed a moderate increase of 5%, while BP-0.5 resulted in only a slight improvement (~1.8%). Overall, the 3% bentonite treatment (BP-3) produced the most favorable outcomes, yielding the highest biomass and vitamin C accumulation (Table 1).

### 3.2. Color Value of Soybean Sprouts

In terms of visual appearance, Hunter color values showed no significant differences in lightness (L*) across treatments (range: 75.59–77.94, all labeled ‘a’), indicating that bentonite treatment did not darken or bleach the sprouts to a degree noticeable by statistical standards. Similarly, redness (a*) values remained unchanged (1.75–1.77, all ‘a’), suggesting no significant shift toward green or red hues. However, significant differences were observed in yellowness (b*), where BP-1 (20.54), BP-0.5 (20.36), and BP-5 (20.21) had significantly higher b* values compared to the control (19.98) and BP-3 (19.95). This subtle increase in yellowness may be perceived as a brighter or fresher appearance, potentially enhancing consumer appeal. Slight increases in b* values are often associated with higher carotenoid content or changes in surface reflectance due to mineral interaction, although this was not directly measured in the current study (Table 2).

### 3.3. Mineral Content

Bentonite pre-treatment influenced the mineral composition of soybean sprouts in varying degrees. Calcium content remained largely stable across treatments, with only slight changes ranging from 1.6% lower to 0.7% higher than the control. Copper content increased notably in BP-0.5 and BP-1 by approximately 20–22%, while BP-3 showed a modest increase of 7.7%, and an unusually high value was observed in BP-5, which likely represents a measurement anomaly. Iron content showed minor fluctuations, decreasing slightly in BP-1 (about 2.9%) and BP-3 (around 9.4%) but increasing in BP-5 by 11%. Potassium levels increased in BP-0.5 (4%) and BP-5 (2.4%) but decreased in BP-1 and BP-3. Magnesium remained relatively constant, with differences within ±1.7%. Manganese content decreased in BP-0.5, BP-1, and BP-3, with the lowest reduction in BP-0.5 (23.3%), while BP-5 was comparable to the control. Sodium content increased in BP-0.5 (+11.2%) and BP-5 (+3.1%) but declined in BP-1 and BP-3. Zinc content decreased gradually with increasing bentonite concentration, reaching a maximum reduction of 31% in BP-5. Phosphorus levels improved considerably with bentonite treatment, rising from 11,600.91 mg/kg in the control to 14,601.41 mg/kg in BP-5 (25.9% higher). Overall, the total mineral content increased with bentonite application, reaching the highest value in BP-5 (11.3% higher than the control), indicating that bentonite can enhance nutrient accumulation in soybean sprouts depending on the element and concentration applied (Table 3).

### 3.4. Isoflavone Content

Bentonite pre-treatment significantly influenced the isoflavone composition of soybean sprouts. Daidzin content increased progressively with treatment, rising by 17.3% in BP-0.5, 28.8% in BP-1, and reaching the highest levels in BP-3 and BP-5 (37.3% and 38.0% higher than the control, respectively). Daidzein remained largely unchanged in BP-0.5 but increased markedly in BP-1 (81%), BP-3 (90.8%), and BP-5 (121%) relative to the control. Genistin content also improved with bentonite treatment, with increases of 10.3%, 37.4%, 61.3%, and 77.5% in BP-0.5, BP-1, BP-3, and BP-5, respectively. Glycitin exhibited a similar trend, rising from 61.28 μg/g in the control to 87.66 μg/g in BP-5, corresponding to a 43% increase. Conversely, glycitein and genistein decreased progressively with bentonite application, with reductions of 14–48% for glycitein and 11–51% for genistein, indicating a shift in the isoflavone profile. Overall, the total isoflavone content increased consistently with bentonite treatment, from 480.24 μg/g in the control to 674.11 μg/g in BP-5, representing a 40.4% enhancement (Table 4). These results suggest that bentonite pre-treatment can selectively enhance glycoside isoflavones while reducing the corresponding aglycones, thereby modifying the isoflavone profile of soybean sprouts.

### 3.5. DPPH, Total Polyphenol and Flavonoid Contents, and SOD-like Activity

Bentonite pre-treatment significantly enhanced the antioxidant properties of soybean sprouts. DPPH radical-scavenging activity increased slightly in BP-0.5 (about 1.2% higher than the control) and showed substantial improvement in BP-1 (approximately 23.6% higher), reaching the highest levels in BP-3 (around 32.6% higher) and BP-5 (about 31.5% higher) compared to the control. Total polyphenol content decreased slightly in BP-0.5 (approximately 12.8% lower than the control) but increased in BP-1 (around 3.1% higher) and further in BP-3 (approximately 17.0% higher) and BP-5 (about 20.5% higher). Total flavonoid content improved across all treatments, rising from 601.50 μg QE/g in the control to levels 9.3%, 16.5%, 25.7%, and 26.4% higher in BP-0.5, BP-1, BP-3, and BP-5, respectively. SOD-like activity also increased progressively, from 25.66% in the control to values 17.3%, 38.1%, 54.6%, and 55.9% higher in BP-0.5, BP-1, BP-3, and BP-5, respectively (Table 5). Overall, bentonite concentrations of 3–5% (BP-3 and BP-5) were most effective in enhancing antioxidant capacity, phenolic accumulation, flavonoid content, and enzymatic defense activity in soybean sprouts.

### 3.6. Free Amino Acid Composition

The free amino acid (FAA) profile of soybean sprouts was significantly affected by the application of bentonite powder (BP) at varying concentrations (0.5%, 1%, 3%, and 5%) compared to the untreated control. Total FAA content ranged from 17.73 to 19.31 mg/g dry weight, with the highest accumulation observed at 3% BP treatment (19.31 mg/g), followed by the control (18.47 mg/g), indicating that moderate bentonite supplementation enhanced amino acid accumulation (Table 6).

#### 3.6.1. Essential Amino Acids (EAAs)

Among EAAs, L-Valine content was significantly increased in all BP treatments, with the highest levels recorded in BP-1 (2.71 mg/g) and BP-0.5 (2.69 mg/g), both significantly higher than the control (2.33 mg/g). A remarkable increase in L-Methionine content was observed in BP-3 (1.20 mg/g) and BP-5 (1.21 mg/g), representing a nearly 7.5-fold increase over the control (0.16 mg/g), indicating a strong induction of sulfur-containing amino acid biosynthesis at higher bentonite levels. L-Lysine, in contrast, showed a declining trend with increasing BP concentration, decreasing from 1.21 mg/g (control) to 0.74–0.76 mg/g under BP-3 and BP-5. L-Leucine and L-Isoleucine showed mild fluctuations, with BP-0.5 resulting in slightly elevated values, while BP-5 led to decreases. The content of L-Phenylalanine peaked in BP-5 (1.96 mg/g), showing a significant increase compared to control (1.77 mg/g). Overall, the EAA sub-total was highest in BP-3 (11.36 mg/g), followed by BP-5 (10.72 mg/g), indicating a stimulatory effect of moderate bentonite concentration on essential amino acid accumulation (Table 6).

#### 3.6.2. Non-Essential Amino Acids (NEAAs)

Among NEAAs, L-Glutamic acid was significantly elevated at BP-3 (0.15 mg/g) and BP-5 (0.16 mg/g), compared to control (0.09 mg/g), while L-Serine declined with higher bentonite levels. L-Alanine showed maximum content at BP-3 (1.71 mg/g), surpassing both control (1.61 mg/g) and other treatments. Notably, proline content decreased progressively with increasing BP concentration, dropping from 0.55 mg/g in control to 0.40 mg/g in BP-5. L-Arginine, on the other hand, was significantly higher in all BP treatments compared to control. The subtotal for NEAAs was slightly reduced in BP-treated groups, with values ranging from 5.15 to 5.47 mg/g, compared to 5.60 mg/g in the control.

#### 3.6.3. Other Amino Acids

The other amino acids category remained relatively unchanged across treatments, except for notable enhancements in γ-aminobutyric acid (GABA), which peaked at BP-3 (0.53 mg/g), significantly higher than control (0.48 mg/g). Ethanolamine showed a decreasing trend with bentonite, and urea was highest in BP-3 (0.77 mg/g). The total of this category remained in a narrow range (2.31–2.48 mg/g), with the highest in BP-3 (Table 6).

## 4. Discussion

Bentonite is a naturally occurring superabsorbent substance that is 2:1 mineral based. Due to its high cation exchange, capacity to absorb and retain water and nutrients, and favorable impacts on raising crop output, this conglomerate of clay minerals has drawn significant interest as a soil supplement [26]. The present study demonstrates that bentonite pre-treatment significantly alters the growth performance, nutritional profile, and functional quality of soybean sprouts, with responses varying according to concentration.

Bentonite application enhanced biomass accumulation in a concentration-dependent manner, with BP-3 yielding the highest increase. This improvement may be linked to the water-holding and ion-exchange properties of bentonite, which help retain moisture and essential ions around the germinating seeds, thereby promoting metabolic activity.

Moisture content was statistically similar among bentonite-treated groups; all treatments showed markedly higher moisture which showed the maintenance of hydration efficiency during germination. Increased vitamin C levels, especially in BP-1 and BP-3, further support the notion that bentonite facilitates oxidative metabolism and ascorbate-associated pathways, consistent with reports that mineral-rich substrates enhance vitamin biosynthesis in sprouts. Hormonal regulation involving auxin and gibberellins may have been indirectly stimulated by the mineral environment [27], contributing to the higher vitamin C content observed at BP-1.

Color parameters play a crucial role in consumer perception and product appeal [28,29]. Colorimetric properties revealed minimal changes in lightness and redness, suggesting that bentonite did not induce surface browning or pigment degradation. However, the significant rise in yellowness (b*) observed in BP-0.5, BP-1, and BP-5 may reflect subtle modifications in carotenoid stability or mineral interactions influencing reflectance. Although not directly measured, this trend aligns with earlier findings linking mineral enrichment to enhanced visual appeal in sprouts. Hunter color analysis showed that lightness (L*) and redness (a*) remained statistically unaffected, preserving the visual appeal of the sprouts, which is a favorable attribute for commercial acceptance.

Mineral composition was strongly affected by bentonite, though responses varied by element. The notable increases in phosphorus, copper, iron (in BP-5), and total mineral accumulation highlight bentonite’s role as a mineral reservoir. The rise in phosphorus is particularly important, as it plays a critical role in energy metabolism, membrane integrity, and sprout vigor. Conversely, reductions in zinc and manganese under higher bentonite levels suggest competitive ion absorption, adsorption on clay surfaces, or altered root uptake pathways. These results indicate that while bentonite can enhance overall mineral density, optimal concentrations are required to prevent antagonistic interactions. The beneficial impacts of bentonite as a mineral element regulator has been demonstrated by Iqbal et al. [16], who reported that the bentonite application at 10 g/kg-1of soil is more beneficial for the wheat crop growth and soil fertility status as compared to the bentonite application at 5 g/kg-1 of soil, and increases the grain yield by 42%, LAI by 20.2%, and Stomatal conductance by up to 13.88%. Similarly, Al-Kinani et al. [30] conducted an experiment investigating the effects of bentonite and compost on soil chemical properties and sorghum growth in desert soil. They reported that bentonite (up to 12%) and compost (up to 80 Mg ha^−1^) significantly improved soil pH, organic matter, CEC, and plant traits like height, leaf area, and chlorophyll content. Elements such as Fe, Cu, K, P, and Na were significantly elevated in various treatments.

The high cation exchange capacity of bentonite likely facilitated nutrient retention during germination, improving sprout mineral density. Notably, Cu spiked over seven-fold in BP-5 (379.05 mg/kg), highlighting bentonite’s enrichment capacity but also raising concerns regarding excessive accumulation and potential toxicity. Iron levels also increased moderately, contributing to improved nutritional value. While Ca and Mg remained unchanged, their consistent availability supports their stabilizing role in cellular processes. The inverse relationship between Cu and Zn may reflect competitive uptake mechanisms, a phenomenon also seen in other mineral-fortified systems [27].

Furthermore, minerals such as Mg, K, and Ca are known to combat hypertension [31], while Fe supports cellular respiration and immune function, and Zn, despite its reduction, plays essential roles in DNA synthesis and antioxidant defense [32]. Bentonite effectively enriched soybean sprouts with essential minerals, especially Fe, Cu, K, and P, though attention is needed to balance mineral ratios to avoid nutritional imbalances.

Isoflavone composition exhibited clear shifts toward glycoside forms (daidzin, genistin, glycitin), with BP-3 and BP-5 showing the highest increases. The concurrent decline of aglycones (daidzein, glycitein, genistein) suggests that bentonite may influence enzymatic hydrolysis or glucosylation pathways during germination. Increased glycosides could be beneficial for shelf stability and functionality, as these forms are generally more water-soluble and stable during processing. The 40.4% increase in total isoflavones highlights bentonite as an effective elicitor for secondary metabolite enrichment. These findings align with previous reports that mineral treatments, such as calcium or illite supplementation, enhance the activity of enzymes involved in isoflavone biosynthesis, possibly including phenylalanine ammonia-lyase and isoflavone synthase [33,34]. Our results are in line with Kim et al. [21], who reported that the Pu-erh tea seed soaking elevated isoflavone content in soybean sprouts. Moreover, it was reported that the increased isoflavone content in bentonite-treated plants strengthens their functional value, given the established antioxidant, anti-inflammatory, and hormonal balance properties of isoflavones [33]. Similarly, a study by Ngueyn [35] developed a bentonite-based composite to enhance topical delivery of orobol, a skin-protective isoflavone.

Antioxidant responses were strongly positive under bentonite treatment, particularly at 3% and 5%. Elevated DPPH activity, phenolic content, flavonoids, and SOD-like activity indicate that bentonite stimulates oxidative stress-related signaling or enhances nutrient availability that drives phenolic biosynthesis. These improvements align with the observed rise in vitamin C and isoflavones, both of which contribute to antioxidant defense in sprouts.

Minerals like potassium and calcium are known to influence polyphenol biosynthesis [31,32,33,34], while bentonite itself has shown antioxidative properties in other systems [36]. Moreover, increased SOD-like activity (40.02% in BP-5) indicates enhanced enzymatic antioxidant capacity, which is critical in neutralizing reactive oxygen species (ROS). Elevated antioxidant capacity protects plants from oxidative damage caused by lipid peroxidation, DNA fragmentation, and protein oxidation, and offers functional food value to consumers by reducing oxidative stress [37,38]. Similarly, Muscolo et al. [39] reported that using sulfur bentonite (SB) enriched with orange residue (OR) or olive pomace (OP) significantly improved the antioxidant and phytochemical profile of red onion. The SB-OR treatment led to the highest increase in polyphenols, sulfur compounds, and thiosulfinates in onion. While Mohammadifard et al. [40] reported that bentonite application improved growth, water retention, and antioxidant response in fenugreek under drought stress. It reduced oxidative damage and stress markers while enhancing chlorophyll content, antioxidant activity, and plant resilience. The benefits of bentonite were most pronounced under severe water deficit conditions. Bentonite treatments, particularly at 3–5%, significantly elevated antioxidant activities in soybean sprouts by increasing polyphenol and flavonoid biosynthesis, contributing to their nutraceutical benefits.

Amino acid profiling further demonstrates that bentonite influences primary metabolism. The substantial increase in essential amino acids—especially methionine, valine, and phenylalanine—suggests stimulation of nitrogen assimilation and protein biosynthesis pathways. The dramatic rise in methionine at higher concentrations indicates enhanced sulfur metabolism, likely facilitated by bentonite’s mineral-exchange properties. Although total NEAAs slightly decreased, certain NEAAs such as alanine, glutamic acid, and arginine increased under BP-3 and BP-5, reflecting selective metabolic shifts. Increased GABA and urea levels in BP-3 may reflect improved stress regulation and nitrogen turnover during sprouting.

The shift toward higher isoflavone glycoside content and reduced aglycone levels suggests that bentonite pre-treatment may influence isoflavone interconversion during germination. This effect may be associated with bentonite’s adsorption capacity and mineral ion exchange, which can modify hydration status, pH, and divalent cation availability, thereby indirectly regulating β-glucosidase activity responsible for glycoside hydrolysis. Similar modulation of isoflavone metabolism by external amendments has been reported during legume sprouting [41,42]

A few caveats and limitations of the present study is that bentonite is a mineral clay with potential impurities or variable composition depending on source, and reproducibility may vary across different bentonite batches or origins. The safety of bentonite manufacture and application needs to be assured with further research to ensure public health. The current study measured compositional and biochemical changes at sprout harvest; however, it did not evaluate bioavailability of the enhanced compounds after consumption (e.g., digestibility, absorption). The sprouting environment (e.g., temperature, light, pH) and potential interactions with bentonite (e.g., pH buffering, ion release) are factors which may influence results. Additionally, higher bentonite concentrations could lead to overaccumulation of certain minerals (e.g., copper), yet no safety assessment was performed, which should be considered in future work to ensure nutritional and toxicological safety.

Collectively, these findings indicate that bentonite functions as both a mineral source and metabolic modulator during sprout development. Moderate concentrations (1–3%) promote balanced improvements in growth, nutrient accumulation, antioxidant capacity, and amino acid biosynthesis, whereas excessive concentration (5%) enhances certain parameters but may negatively affect others due to ion competition or metabolic stress. The study highlights bentonite as a low-cost, environmentally friendly, and effective enhancer for producing nutritionally enriched soybean sprouts. Further work should explore field experiments and molecular mechanisms underlying these responses and evaluate bentonite’s long-term safety and applicability in commercial sprout production as well as on field production that may enhance yield and oil production.

## 5. Conclusions

Given the global interest in nutrient-rich, plant-based foods, incorporating bentonite pre-treatment in sprout production could contribute to improved food quality and functional-food development. Further research—particularly into bioavailability and the mechanisms underlying the observed effects—would strengthen the case for practical application and possible scale-up.

## Figures and Tables

**Table 1:** Effect of different concentrations of bentonite treatment on the yield, moisture content, and vitamin C content of soybean sprouts cultivated for 6 days.

Sample	Total Weight (g)	Moisture (%)	Vitamin C (mg/100 g Fresh Weight)
Control	5675 ± 25 e (100.0%)	86.99 ± 1.05 a	16.01 ± 0.30 c
BP-0.5	5799 ± 16 d (102.2%)	87.20 ± 0.81 a	16.3 ± 0.33 bc
BP-1	6259 ± 19 b (110.3%)	87.41 ± 0.38 a	18.80 ± 0.21 a
BP-3	6588 ± 21 a (116.1%)	86.98 ± 1.02 a	18.91 ± 0.19 a
BP-5	5913 ± 20 c (104.2%)	87.31 ± 0.59 a	16.81 ± 0.03 b

Control: soybean seeds soaked in tap water for 8 h; BP-0.5, BP-1, BP-3, and BP-5: seeds soaked in tap water containing 0.5, 1.0, 3.0, and 5.0% (*w*/*v*) bentonite powder, respectively, for 8 h. Values are expressed as mean ± standard deviation of three replicates. Different letters in the same column indicate significant differences (*p* < 0.05, Tukey’s test).

**Table 2:** Hunter’s color values of soybean sprouts grown after different concentrations of bentonite treatment.

Sample	Color Value		
	L* (Lightness)	a* (Redness)	b* (Yellowness)
Control	77.94 ± 0.03 a	1.77 ± 0.03 a	19.98 ± 0.02 b
BP-0.5	76.41 ± 016 a	1.75 ± 0.02 a	20.36 ± 0.05 a
BP-1	76.60 ± 0.27 a	1.75 ± 0.04 a	20.54 ± 0.23 a
BP-3	75.59 ± 0.82 a	1.75 ± 0.02 a	19.95 ± 0.05 b
BP-5	76.00 ± 0.45 a	1.76 ± 0.03 a	20.21 ± 0.95 a

Sample abbreviations are defined in Table 1. L* represents lightness (100, white; 0, black); a*, redness (−, green; +, red); b*, yellowness (−, blue; +, yellow). Values are expressed as mean ± standard deviation of three replicates. Values followed by different letters in the same column are significantly different (*p* < 0.05, Tukey test).

**Table 3:** Mineral contents (mg/kg of dry weight) of soybean sprouts cultivated after different concentrations of bentonite treatment.

Element	Control	BP-0.5	BP-1	BP-3	BP-5
Ca (mg/kg)	1467.71 ± 16.63 a	1477.61 ± 38.23 a	1444.61 ± 14.57 a	1469.45 ± 30.84 a	1445.75 ± 24.59 a
Cu (mg/kg)	52.78 ± 0.02 d	63.33 ± 0.81 b	64.41 ± 1.56 b	56.84 ± 0.06 c	79.05 ± 1.23 a
Fe (mg/kg)	10.15 ± 0.07 c	10.34 ± 0.01 b	9.85 ± 0.09 d	9.19 ± 0.06 e	11.26 ± 0.07 a
K (mg/kg)	6956.60 ± 31.13 c	7240.91 ± 34.42 a	6437.51 ± 85.80 d	6923.94 ± 74.66 c	7122.12 ± 56.10 b
Mg (mg/kg)	5987.86 ± 90.23 a	6087.36 ± 51.89 a	5928.03 ± 15.47 a	5899.60 ± 10.04 b	5992.42 ± 4.70 b
Mn (mg/kg)	52.88 ± 0.03 b	40.59 ± 0.05 e	49.07 ± 0.43 c	48.51 ± 0.02 d	52.99 ± 0.05 a
Na (mg/kg)	2418.38 ± 2.64 c	2688.37 ± 1.45 a	2123.66 ± 2.38 e	2283.02 ± 1.24 d	2494.18 ± 4.50 b
Zn (mg/kg)	58.12 ± 0.14 a	45.66 ± 0.05 b	45.03 ± 0.07 c	41.70 ± 0.22 d	40.12 ± 0.03 e
P (mg/kg)	11,600.91 ± 178.51 e	12,685.30 ± 70.91 c	12,322.41 ± 105.42 d	13,103.40 ± 121.40 b	14,601.41 ± 103.51 a
Total	28,605.39	30,339.46	28,424.58	29,835.65	31,839.30

Sample abbreviations are defined in Table 1. Values are expressed as mean ± standard deviation of three replicates. Values followed by different letters in the same column are significantly different (*p* < 0.05, Tukey test).

**Table 4:** Isoflavone content (mg/kg dry weight) of soybean sprouts cultivated after different concentrations of bentonite treatment.

Isoflavone	Sample				
	Control	BP-0.5	BP-1	BP-3	BP-5
Daidzin	231.99 ± 3.22 d	272.32 ± 3.12 c	298.76 ± 4.92 b	318.51 ± 3.12 a	320.22 ± 3.98 a
Daidzein	12.11 ± 0.81 d	12.09 ± 1.20 d	21.91 ± 0.97 c	23.11 ± 1.32 b	26.76 ± 0.88 a
Genistin	119.22 ± 3.11 e	131.52 ± 4.02 e	163.77 ± 1.29 c	192.33 ± 3.11 b	211.62 ± 2.39 a
Glycitin	61.28 ± 1.62 e	65.51 ± 2.09 d	70.53 ± 1.30 c	75.22 ± 1.51 b	87.66 ± 2.09 a
Glycitein	15.37 ± 0.22 a	13.20 ± 0.21 b	11.36 ± 0.28 c	8.02 ± 0.61 d	7.98 ± 0.55 d
Genistein	40.27 ± 1.92 a	35.6 ± 0.41 b	31.98 ± 1.2 c	26.23 ± 1.69 d	19.87 ± 2.31 e
Total	480.24	530.30	596.31	643.42	674.11

Sample abbreviations are defined in Table 1. Values are expressed as mean ± standard deviation of three replicates. Values followed by different letters in the same column are significantly different (*p* < 0.05, Tukey test).

**Table 5:** 1,1-diphenyl-2-picrylhydrazyl (DPPH), total polyphenol and flavonoid contents, and superoxide dismutase (SOD)-like activity of soybean sprouts treated with different concentrations of bentonite treatment.

Sample	DPPH (% Inhibition)	Total Polyphenol (μg GAE/g)	Total Flavonoid (μg QE/g)	SOD-like Activity (% Inhibition)
Control	65.31 ± 1.62 c	483.16 ± 10.26 c	601.50 ± 7.22 d	25.66 ± 1.09 d
BP-0.5	66.12 ± 1.51 c	421.31 ± 12.00 d	657.44 ± 5.92 c	30.11 ± 0.45 c
BP-1	80.69 ± 0.60 b	498.545 ± 9.66 c	701.27 ± 9.12 b	35.44 ± 1.29 b
BP-3	86.61 ± 0.98 a	565.31 ± 7.39 b	756.15 ± 5.15 a	39.68 ± 1.04 a
BP-5	85.91 ± 1.50 a	582.12 ± 6.78 a	760.22 ± 4.98 a	40.02 ± 0.98 a

Sample abbreviations are defined in Table 1. Values are expressed as mean ± standard deviation of three replicates. Values followed by different letters in the same column are significantly different (*p* < 0.05, Tukey test).

**Table 6:** Free amino acid composition (mg/g of dry weight) of soybean sprouts cultivated after different concentrations of bentonite treatment.

Amino Acid	Sample				
	Control	BP-0.5	BP-1	BP-3	BP-5
Essential Amino Acid					
L-Threonine	1.61 ± 0.01 a	1.35 ± 0.01 d	1.41 ± 0.02 c	1.51 ± 0.02 b	1.21 ± 0.02 e
L-Valine	2.33 ± 0.02 c	2.69 ± 0.02 a	2.71 ± 0.03 a	2.62 ± 0.01 b	2.22 ± 0.01 d
L-Methionine	0.16 ± 0.01 b	0.15 ± 0.01 b	0.16 ± 0.01 b	1.20 ± 0.02 a	1.21 ± 0.03 a
L-Isoleucine	1.09 ± 0.03 b	1.18 ± 0.02 a	1.06 ± 0.02 b	1.11 ± 0.03 b	1.03 ± 0.01 c
L-Leucine	0.55 ± 0.01 b	0.62 ± 0.02 a	0.53 ± 0.01 bc	0.51 ± 0.02 c	0.50 ± 0.02 c
L-Phenylalanine	1.77 ± 0.02 c	1.70 ± 0.01 d	1.69 ± 0.02 d	1.82 ± 0.02 b	1.96 ± 0.02 a
L-Lysine	1.21 ± 0.01 a	0.94 ± 0.01 b	0.89 ± 0.02 c	074 ± 0.02 d	0.76 ± 0.021 d
Ll-Histidine	1.71 ± 0.02 c	1.70 ± 0.01 c	1.82 ± 0.02 b	1.86 ± 0.01 a	1.83 ± 0.02 b
Sub-total	10.43	10.33	10.27	11.36	10.72
Non-essential Amino Acid					
L-Asparitic acid	0.35 ± 0.01 a	0.34 ± 0.01 a	0.30 ± 0.02 b	0.35 ± 0.01 a	0.36 ± 0.02 a
L-Serine	1.92 ± 0.02 a	1.91 ± 0.04 a	1.69 ± 0.02 b	1.59 ± 0.02 c	1.63 ± 0.02 c
L-Glutamic acid	0.09 ± 0.01 b	0.06 ± 0.02 c	0.09 ± 0.01 b	0.15 ± 0.01 a	0.16 ± 0.02 a
Glycine	0.16 ± 0.01 b	0.15 ± 0.02 b	0.15 ± 0.01 b	0.19 ± 0.01 a	0.18 ± 0.01 ab
L-Alanine	1.61 ± 0.02 b	1.31 ± 0.03 d	1.45 ± 0.01 c	1.71 ± 0.02 a	1.59 ± 0.02 b
L-Tyrosine	0.13 ± 0.02 a	0.11 ± 0.02 a	0.12 ± 0.01 a	0.12 ± 0.01 a	0.13 ± 0.02 a
L-Arginine	0.79 ± 0.01 B	0.88 ± 0.02 a	0.89 ± 0.01 a	0.91 ± 0.01 a	0.89 ± 0.01 a
Proline	0.55 ± 0.01 a	0.47 ± 0.02 b	0.46 ± 0.01 b	0.45 ± 0.02 b	0.40 ± 0.01 c
Sub-total	5.60	5.23	5.15	5.47	5.34
Other Amino Acid					
*O*-Phospho-L-serine	0.14 ± 0.01 a	0.15 ± 0.02 a	0.14 ± 0.01 a	0.14 ± 0.01 a	0.13 ± 0.01 a
Taurine	ND	ND	ND	ND	ND
*O*-Phospho ethanol amine	ND	ND	ND	ND	ND
Urea	0.75 ± 0.02 ab	0.74 ± 0.01 b	0.65 ± 0.02 c	0.77 ± 0.01 a	0.74 ± 0.02 ab
L-Sarcosine	0.03 ± 0.01 ab	0.01 ± 0.01 b	0.02 ± 0.01 ab	0.04 ± 0.01 a	0.04 ± 0.01 a
L-α-Amino asipic acid	0.17 ± 0.02 ab	0.16 ± 0.01 ab	0.16 ± 0.01 ab	0.17 ± 0.01 a	0.14 ± 0.01 b
L-Citrulline	0.04 ± 0.01 a	0.04 ± 0.01 a	0.05 ± 0.01 a	0.04 ± 0.01 a	0.05 ± 0.01 a
L-α-Amino-n-butyric acid	0.08 ± 0.01 a	0.09 ± 0.01 a	0.07 ± 0.02 a	0.08 ± 0.01 a	0.08 ± 0.01 a
L-Cystine	0.07 ± 0.01 a	0.07 ± 0.01 a	0.08 ± 0.01 a	0.09 ± 0.01 a	0.08 ± 0.01 a
Cystathionine	0.02 ± 0.01 a	0.03 ± 0.01 a	0.03 ± 0.01 a	0.02 ± 0.01 a	0.03 ± 0.01 a
β-Alanine	0.31 ± 0.02 a	0.32 ± 0.01 a	0.30 ± 0.02 a	0.31 ± 0.01 a	0.32 ± 0.01 a
D,L-β-Amino isobutyric acid	0.11 ± 0.1 a	0.10 ± 0.02 a	0.12 ± 0.01 a	0.11 ± 0.01 a	0.10 ± 0.02 a
γ-Amino-n-butyric acid	0.48 ± 0.02 bc	0.47 ± 0.01 c	0.50 ± 0.01 b	0.53 ± 0.12 a	0.47 ± 0.01 c
Ethanolamin	0.28 ± 0.02 a	0.24 ± 0.01 b	0.25 ± 0.01 b	0.21 ± 0.01 c	0.22 ± 0.01 c
Hydroxylysine	ND	ND	ND	ND	ND
L-Ornithine	0.02 ± 0.01 a	0.02 ± 0.01 a	0.01 ± 0.01 a	0.02 ± 0.01 a	0.01 ± 0.01 a
1-Methyl-L-histidine	ND	ND	ND	ND	ND
3-Methyl-L-histidine	ND	ND	ND	ND	ND
L-Anserine	ND	ND	ND	ND	ND
L-Carnosine	ND	ND	ND	ND	ND
Hydroxy proline	0.08 ± 0.02 a	0.06 ± 0.02 a	0.07 ± 0.01 a	0.09 ± 0.02 a	0.08 ± 0.02 a
Sub-total	2.44	2.35	2.31	2.48	2.36
Total Free Amino Acid	18.47	17.91	17.73	19.31	18.42

Sample abbreviations are defined in Table 1. Values are expressed as mean ± standard deviation of three replicates. Values followed by different letters in the same column are significantly different (*p* < 0.05, Tukey test). Where ND represents (non-detectable).

**Figure A1:**
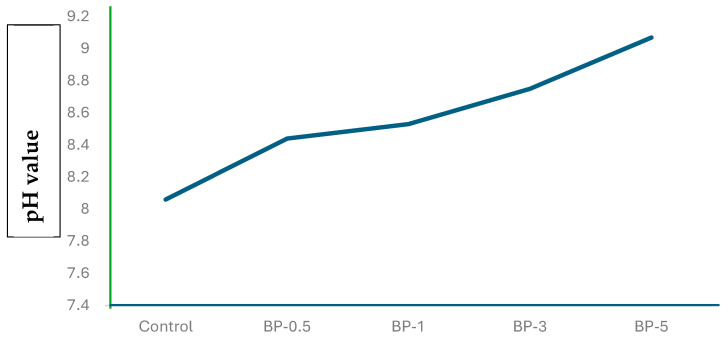
pH value of soaking media containing different concentrations of bentonite.

## Data Availability

The original contributions presented in the study are included in the article, further inquiries can be directed to the corresponding authors.

## Appendix A

Bentonite composition: broccoli sprout powder standardized for sulforaphane potential, glucosinolates and glucoraphanin, broken-cell-wall chlorella, calcium D-glucarate, dandelion root and leaf, PE 4:1, yellow dock root PE 4:1, burdock root PE 4:1, DIM (di-indolyl-methane), nettle leaf PE 4:1. Additional ingredients: Non-GMO, corn-free, gluten-free, maltodextrin made from Ipomoea batatas.

## Appendix B

The DPPH radical scavenging activity was calculated by using the following equation:

DPPH radical scavenging activity (%) = [1 − (B − B_0_)/(A − A_0_)] × 100

where A = Absorbance of the sample with DPPH, A_0_ = Absorbance of the sample blank (sample without DPPH), B = Absorbance of the control (DPPH solution without sample), B_0_ = Absorbance of the control blank (solvent without DPPH)

The SOD-like activity and the standard formula for SOD-like activity (%) based on inhibition of absorbance were calculated using the following equation:

$$\mathrm{SOD}-\mathrm{like}\;\mathrm{activity}\;(\%)=\left(\frac{S_{0}-S}{S_{0}}\right)\times 100$$

where S_0_ = Change in absorbance measured with Tris–HCl buffer instead of sample extract (control), S = Change in absorbance measured with the sample extract.
